# Myostatin/Smad3 in Spinal Muscular Atrophy: Biomarkers of Disease Severity and Treatment Response

**DOI:** 10.1002/acn3.70564

**Published:** 2026-10-26

**Authors:** Giorgia Riolo, Claudia Dosi, Stefano Parravicini, Riccardo Zanin, Marta Cheli, Eleonora Giacopuzzi Grigoli, Amalia Canciello, Francesca Andreetta, Riccardo Masson, Silvia Bonanno, Stefania Marcuzzo, Lorenzo Maggi

**Affiliations:** ^1^ Neurology 4—Neuroimmunology and Neuromuscular Diseases Unit Fondazione IRCCS Istituto Neurologico Carlo Besta Milan Italy; ^2^ Ph.D. Program in Pharmacological Biomolecular Sciences, Experimental and Clinical University of Milan Milan Italy; ^3^ Developmental Neurology Unit Fondazione IRCCS Istituto Neurologico Carlo Besta Milan Italy; ^4^ Brain‐Targeted Nanotechnologies (BraiNs) Lab Joint Research Platform—Fondazione IRCCS Istituto Neurologico Carlo Besta Milan Italy

**Keywords:** disease‐modifying therapies, muscle homeostasis, Myostatin, non‐invasive biomarkers, patient‐specific response, Smad3, spinal muscular atrophy

## Abstract

**Objective:**

Given the role of Myostatin (MSTN) in muscle homeostasis and its modulation by current spinal muscular atrophy (SMA) therapies, we investigated serum MSTN and, for the first time, its downstream effector Smad3 as candidate non‐invasive biomarkers in SMA patients undergoing disease‐modifying therapies (DMTs).

**Methods:**

The study included pediatric and adult SMA patients receiving DMTs (Nusinersen = 15; Risdiplam = 15), and participants in anti‐MSTN clinical trials (*n* = 7), whose data were analyzed only at baseline. Serum MSTN and Smad3 levels were quantified by ELISA at baseline, 6 and 14 months post‐treatment in pediatric and adult SMA patients, respectively, considering the slower DMTs effects in adulthood.

**Results:**

Baseline MSTN levels were significantly (*p*‐value < 0.05) higher in: males (915.0 ± 592.5 pg/mL) vs. females (593.4 ± 231.4 pg/mL); SMA III (952.2 ± 598.9 pg/mL) vs. II (541.1 ± 326.4 pg/mL), and walkers (1047.6 ± 548.9 pg/mL) vs. non‐sitters (374.6 ± 188.5 pg/mL). Smad3 showed no correlation and remained stable during treatment. In adults MSTN levels positively correlated with Hammersmith Functional Motor Scale Expanded (HFMSE) at baseline (*p* = 0.01) and post‐treatment (*p* = 0.003). Smad3 levels negatively correlated with baseline HFMSE (*p* = 0.03) and post‐treatment Revised Upper Limb Module scores (*p* = 0.03). In pediatric patients, MSTN fold change (i.e., post−/pre‐therapy ratio) at baseline negatively correlated with HFMSE changes (*p* = 0.008).

**Interpretation:**

MSTN levels were associated with clinical phenotype and motor function, supporting its role as a candidate biomarker of SMA severity in adult and pediatric patients. Our findings also suggest that MSTN may serve as a potential dynamic biomarker of disease progression in pediatric patients, regardless of treatment. Further validation in independent cohorts is warranted.

## 1 Introduction

Spinal muscular atrophy (SMA) is a rare, genetic neuromuscular condition causing progressive muscle wasting (atrophy) and weakness leading to loss of movement. Overall prevalence of SMA ranges between 1 and 2 per 100,000 people, with a frequency of 1 in 11,000 births [1, 2]. Historically, SMA has been subdivided into five subtypes (SMA 0‐IV) based on age at onset, ranging from in utero to adulthood and motor milestones achieved, from absent or severely reduced motor function to very mild phenotypes [3]. Approximately 96% of SMA cases are caused by mutations or homozygous deletions in the *SMN1* gene, located on chromosome 5q13.2. *SMN1* encodes the majority of functional full‐length (FL) survival motor neuron (SMN) protein, which is critical for motor neuron viability. There is a nearly identical *SMN2* gene peculiar to humans, present in the same region of chromosome 5. The difference between *SMN1* and *SMN2* is a C > T substitution in exon 7 of *SMN2*, which alters RNA splicing. Consequently, the majority of *SMN2* transcripts lack exon 7, producing a truncated, non‐functional protein. Only about 10%–15% of *SMN2* transcript is alternatively spliced to include exon 7, leading to the production of a small amount of full‐length SMN protein, which is insufficient to prevent disease [4, 5]. Complete absence of SMN is embryonically lethal, while diminished SMN content causes anterior horn cells to degenerate, ultimately resulting in motor neuron loss and subsequent skeletal muscle atrophy and weakness. Although the *SMN2* gene can express small amounts of the full‐length *SMN* transcript, the number of *SMN2* copies, ranging from 0 to ≥ 4, represents a disease modifier where a higher *SMN2* copy number is usually associated with a milder phenotype [6].

In the last decade, substantial progress has been made in SMA treatment, culminating in the approval of 3 therapies. These therapies act by upregulating SMN protein in affected individuals, either through the enhancement of exon 7 inclusion in mature mRNA, as determined by risdiplam (Evrysdi) and nusinersen (Spinraza), or via AAV9‐mediated gene replacement therapy onasemnogene abeparvovec (Zolgensma) [7]. Furthermore, given the role of Myostatin (MSTN), a protein functioning as a potent negative regulator of skeletal muscle mass, several MSTN inhibitors are currently being investigated as potential therapeutic agents in SMA. Notably, MSTN‐targeting antibodies have emerged as particularly promising, due to their ability to act at different stages of MSTN expression [8]. For instance, apitegromab selectively binds to the latent form of MSTN, stabilizing its conformation and thereby preventing access to proteolytic cleavage sites required for activation [9]. This approach, as reported in a Phase 3 trial, improved motor function in nonambulatory SMA II and SMA III patients and was generally well tolerated, supporting the therapeutic potential of muscle‐targeted strategies in SMA [10]. Similarly, GYM−329/emugrobart's ability to target latent MSTN and block its conversion into the mature, bioactive form was investigated in a recent clinical trial [11]. In contrast, taldefgrobep is directed against mature MSTN, inhibiting its interaction with activin Type II receptors, thus promoting skeletal muscle growth and function [12].

In fact, like other members of the TGF‐β family, MSTN is secreted as an inactive precursor and becomes active following cleavage of its propeptides. Upon activation, mature MSTN is released into the extracellular space, where it binds to activin receptors (ActRIIA/B) on the surface of myoblasts, initiating a complex signaling cascade, consisting in the following steps: (i) receptor dimerization; (ii) activation of activin‐like kinases (ALK4 or ALK5); (iii) phosphorylation of Smad 2 and Smad3, which form the Smad 4 complex; (iv) Smad 4 translocation to the nucleus; (v) suppression of genes involved in myogenesis (e.g., Pax7, MyoD, MyoG, and MyHC); and (vi) increased expression of genes associated with muscle atrophy (e.g., Atrogin1 and MuRF1) [13, 14].

Of note, serum MSTN levels are reduced in SMA patients compared to healthy subjects, reflecting the extent of motor neuron degeneration [15]. Nevertheless, in walker SMA III, the greater preservation of muscle mass is associated with higher circulating MSTN levels than in non‐walker SMA III patients, although still lower than in healthy individuals [16].

With the availability of disease‐modifying therapies (DMTs) and on‐going clinical trials of emerging treatments, understanding the SMA natural history and identifying non‐invasive biomarkers to monitor disease progression and patient‐specific response to treatments represent crucial medical needs. Efforts to identify a non‐invasive biomarker for SMA have culminated in a number of putative molecular markers [17]. Among these, neurofilaments (NFs) have been proven to be a robust prognostic, disease progression and pharmacodynamic biomarker, suitable to monitor neurodegeneration, particularly in SMA children, although their role in SMA patients in adult age is still debated [18]. However, most of the studies on biomarkers are limited to small and monocentric cohorts and there is currently no international agreement on their application in routine clinical practice.

In this perspective, considering the role of MSTN in muscle homeostasis and taking into account that MSTN is a direct or indirect target of current therapeutic approaches for SMA, we aimed to explore the role of serum MSTN as a non‐invasive biomarker for monitoring disease progression and therapeutic response in pediatric and adult SMA patients. Along with MSTN, Smad3 levels were also analyzed in the serum from the same patients, since MSTN exerts its effects through the activation of the Smad3 transcription factor, which translocates to the nucleus to regulate genes involved in muscle growth inhibition.

## 2 Methods

### 2.1 Patients and Biological Samples

A cohort of 37 clinically and genetically defined pediatric and adult SMA patients followed at the Fondazione IRCCS Istituto Neurologico Carlo Besta (Milan, Italy) were involved in the study. The cohort included patients treated with approved DMTs as monotherapy, nusinersen (*n* = 15 patients) and risdiplam (*n* = 15 patients), and participants enrolled in anti‐MSTN pharmacological clinical trials (*n* = 7).

Among the entire cohort, 8 patients were analyzed only at baseline. Specifically, 7 patients enrolled in anti‐MSTN clinical trials, for whom follow‐up samples were unavailable, were therefore excluded from post‐treatment MSTN and Smad3 expression analyses because they were still actively participating in the clinical trials at the time of this study; 1 risdiplam‐treated patient who underwent a surgical procedure during the treatment period, hence not reliable for clinical change evaluation.

All patients provided serum samples and underwent clinical assessments. Specifically, serum samples were obtained from peripheral blood immediately before first infusion (T0, baseline), after 6 months of treatment (T6) in pediatric patients and after 14 months (T14) in adult SMA patients, considering the smaller and slower effect of DMTs in the adult age. Serum Separator Tubes were used for blood collection and centrifuged at 3000 rpm for 10 min at 4°C; aliquots of the isolated serum were promptly stored at −80°C in the Biobank of Fondazione IRCCS Istituto Neurologico Carlo Besta until use.

### 2.2 Ethics

The study was performed in accordance with the ethical standards of the Declaration of Helsinki. The investigation and use of patients' data for research purposes were approved by the Fondazione IRCCS Istituto Neurologico Carlo Besta research ethical committee (Project identification code 92/2019, 16 January 2019). Patients or their parents signed an informed consent form.

### 2.3 Clinical Assessments

Clinical, motor function and safety data were collected at each time point. According to the international recommendations on SMA standard of care, the following primary outcome measures were assessed by trained evaluators [19]: the Hammersmith Functional Motor Scale Expanded (HFMSE), consisting of 33 items, each scored from 0 to 2, up to a maximum of 66 points with higher scores indicating better motor performance; Revised Upper Limb Module (RULM), including 20 items with a maximum score of 37 and higher scores indicating better upper limb motor function. An improvement of ≥ 3 points on the HFMSE or ≥ 2 points on the RULM from baseline was considered clinically meaningful [20, 21]. We define as “responders” those patients with a clinically meaningful change of HFMSE and/or RULM.

### 2.4 Enzyme‐Linked Immunosorbent Assay (ELISA)

The Human GDF‐8/latent MSTN and total Smad3 (Novus Biologicals, Littleton, CO, USA) ELISA kits (Colorimetric) were used to measure serum concentration of MSTN and Smad3 respectively. We used assay protocol and sample dilution in accordance with the manufacturers' instructions.

### 2.5 Statistical Analysis

Non‐parametric distributed data were analyzed by the Kruskal–Wallis test with Dunn's post hoc test for comparisons among groups, or by the Mann–Whitney test for comparison between 2 groups. Fold change (FC) was calculated for each group as the ratio between post‐treatment and baseline values. In this study, a common cut‐off value of 1 was used to distinguish between increased (FC > 1), decreased (FC < 1), and unchanged (FC = 1) protein values. The use of a FC threshold of 1 is appropriate and widely accepted when the goal is to qualitatively classify increases or decreases in protein levels rather than to identify only substantial changes [22, 23]. Pearson or Spearman correlation coefficients were evaluated in correlation analyses. A *p*‐value < 0.05 was considered statistically significant. GraphPad Prism v 8.0 software was used for data elaboration and statistical analyses.

## 3 Results

### 3.1 SMA Patient Cohort and Response to Therapy

Median age at onset of the 37 included patients (18 males and 19 females) was 2 (range 0.6–26) years, while median age at baseline was 17 (range 6–50) years, among which 19 (51.4%) were pediatrics (age < 18 years) and 18 (48.6%) adults.

The cohort included 1 (2.7%) SMA I, 19 (51.4%) SMA II, and 17 (45.9%) SMA III, classified at baseline according to the motor function into: non‐sitters (*n* = 6, unable to sit independently), sitters (*n* = 15, able to sit but not walk independently), and walkers (*n* = 16, able to walk independently).

Patients' clinical features and motor function assessments at baseline (T0) and after treatment, T6 in pediatric and T14 in adult SMA patients, are shown in Tables 1 and 2. Particularly, 6/11 (54.5%) pediatric and 5/18 (27.8%) adult SMA patients were classified as responders. Among pediatric responders, 6/7 were SMA II (1 non‐sitter and 5 sitters), and 1/7 was SMA III walker. All adult responders were SMA III walkers. Of note, 1 pediatric SMA III walker and 4 adult SMA III walker patients did not meet the responder criteria, due to a ceiling effect having consistently high baseline scores on both scales.

**TABLE 1:** Clinical features and motor function evaluations of SMA pediatric patients.

Patient	Gender	SMA type	SMN2 copy number	AGE at ONSET (Yr)	AGE at 1st infusion (Yr)	Treatment	T0 motor milestone	HFMSE at T0	HFMSE at T6	ΔHFMSE CMC	RULM at T0	RULM at T6	ΔRULM CMC
Pt 01	F	II	3	1	7	Nusinersen	Sitter	12	14	2	14	16	2
Pt 02	F	II	3	1	7	Nusinersen	Sitter	8	9	1	13	10	−3
Pt 03	F	II	3	1	10	Nusinersen	Sitter	7	7	0	11	14	3
Pt 04	M	II	3	2	8	Nusinersen	Sitter	13	11	−2	23	21	−2
Pt 05	M	II	3	2	7	Nusinersen	Sitter	14	15	1	20	16	−4
Pt 06	M	III	3	6	9	Nusinersen	Walker	57	61	4	37	37	0
Pt 07	F	II	3	6	11	Risdiplam	Sitter	1	2	1	7	10	3
Pt 08	F	II	3	7	9	Risdiplam	Sitter	14	13	−1	16	18	2
Pt 09	F	II	3	7	9	Risdiplam	Sitter	7	6	−1	14	14	0
Pt 10	M	III	4	9	17	Risdiplam	Walker	63	63	0	37	37	0
Pt 11	M	II	3	7	12	Risdiplam	Sitter	3	7	4	15	16	1
Pt 12^a^	M	II	2	2	8	Risdiplam	Non sitter	10	2	NA	10	12	2
Pt 13	M	II	3	1	12	Anti‐MSTN clinical trial	Non sitter	15	NA	NA	32	NA	NA
Pt 14	M	II	3	1,5	10	Anti‐MSTN clinical trial	Non sitter	22	NA	NA	37	NA	NA
Pt 15	F	II	3	1,5	10	Anti‐MSTN clinical trial	Non sitter	33	NA	NA	26	NA	NA
Pt 16	M	III	3	1,5	11	Anti‐MSTN clinical trial	Walker	51	NA	NA	36	NA	NA
Pt 17	F	I	2	NA	6	Anti‐MSTN clinical trial	Sitter	27	NA	NA	18	NA	NA
Pt 18	F	III	4	2	6	Anti‐MSTN clinical trial	Walker	63	NA	NA	33	NA	NA
Pt 19	M	II	3	0,6	9	Anti‐MSTN clinical trial	Sitter	NA	NA	NA	30	NA	NA

*Note:* T0 before and T6 after treatment.

Abbreviation: CMC, clinically meaningful change.

^a^ Patient underwent surgery during treatment; data used for baseline analyses only, due to unreliable clinical change evaluation.

**TABLE 2:** Clinical features and motor function evaluations of SMA adult patients.

Patient	Gender	SMA type	SMN2 copy number	AGE at ONSET (Yr)	AGE at 1st infusion (Yr)	Treatment	T0 motor milestone	HFMSE at T0	HFMSE at T14	ΔHFMSE CMC	RULM at T0	RULM at T14	ΔRULM CMC
Pt 20	F	III	4	2	39	Nusinersen	Sitter	24	24	0	26	26	0
Pt 21	F	III	4	3	25	Nusinersen	Walker	54	57	3	37	36	−1
Pt 22	F	III	4	3	34	Nusinersen	Walker	53	55	2	37	37	0
Pt 23	M	III	4	3	50	Nusinersen	Walker	44	47	3	29	27	2
Pt 24	M	III	4	12	38	Nusinersen	Walker	48	48	0	37	37	0
Pt 25	M	III	3	12	24	Nusinersen	Walker	63	64	1	37	37	0
Pt 26	F	III	3	4	28	Nusinersen	Walker	38	44	6	24	24	0
Pt 27	F	III	4	2	31	Nusinersen	Walker	54	55	1	37	37	0
Pt 28	M	II	3	1	39	Nusinersen	Non sitter	0	0	0	0	0	0
Pt 29	F	II	3	12	44	Risdiplam	Sitter	5	5	0	18	18	0
Pt 30	F	II	3	1	40	Risdiplam	Sitter	2	2	0	10	10	0
Pt 31	F	II	3	2	35	Risdiplam	Sitter	0	0	0	14	14	0
Pt 32	M	III	4	15	31	Risdiplam	Walker	55	56	1	35	35	0
Pt 33	M	III	4	1	21	Risdiplam	Walker	41	48	7	31	30	−1
Pt 34	F	III	4	8	42	Risdiplam	Walker	45	45	0	37	37	0
Pt 35	M	III	4	26	46	Risdiplam	Walker	61	64	3	37	37	0
Pt 36	F	III	4	2	34	Risdiplam	Walker	55	55	0	37	37	0
Pt 37	M	II	3	1	41	Risdiplam	Non sitter	0	0	0	0	0	0
Pt 34	F	III	4	8	42	Risdiplam	Walker	45	45	0	37	37	0

*Note:* T0 before and T14 after treatment.

Abbreviation: CMC, clinically meaningful change.

### 3.2 Basal Serum Levels of MSTN and Smad3

We investigated whether baseline serum MSTN and Smad3 levels were influenced by age, gender, SMA type, *SMN2* copy number, disease duration and motor function status. Statistical analysis revealed significantly higher levels of MSTN in: male (mean ± SD, 915.0 ± 592.5 pg/mL) compared to female (mean ± SD, 593.4 ± 231.4 pg/mL) SMA patients (*p* = 0.03, Figure 1A); SMA III (mean ± SD, 952.2 ± 598.9 pg/mL) compared to SMA II (mean ± SD, 541.1 ± 326.4 pg/mL) (*p* = 0.04, Figure 1B); walkers (mean ± SD, 1047.6 ± 548.9 pg/mL) compared to non‐sitter (mean ± SD, 374.6 ± 188.5 pg/mL) SMA patients (*p* = 0.003, Figure 1C). No significant sex‐related differences were observed in HFMSE or RULM scores (data not shown).

**FIGURE 1:**
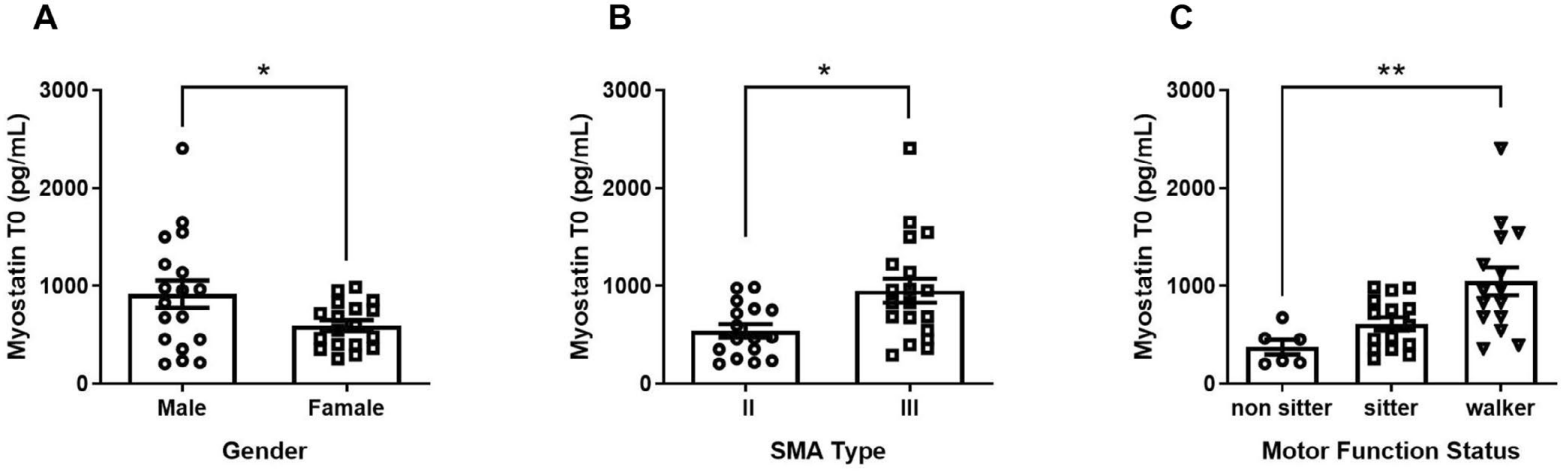
The Wilcoxon–Mann–Whitney and Kruskal–Wallis tests were performed to evaluate differences in serum MSTN levels based on gender (A), SMA type (B), and motor function status (C) at baseline (T0). *p < 0.05, **p < 0.01. Data were screened for outliers; one extreme value (> 3 SD from the mean) was excluded from the analysis.

Conversely, no significant differences in serum Smad3 levels were detected according to gender, SMA type and motor function status. Moreover, serum MSTN and Smad3 levels did not differ significantly among groups stratified by *SMN2* copy number; nor were they associated with age at baseline and disease duration (data not shown).

Of note, the exclusion of patients involved in anti‐MSTN clinical trials (*n* = 7) and the risdiplam‐treated patient did not affect the main baseline findings, indicating that the observed associations were not driven by this specific subgroup.

At baseline, creatine phosphokinase (CK) levels were available for 25 patients (13 pediatric and 12 adult patients) and showed a significant correlation with MSTN levels (*r* = 0.44, *p* = 0.03). When pediatric and adult patients were analyzed separately, this correlation remained significant only in the adult subgroup (*r* = 0.59, *p* = 0.04). Baseline body weight was available for 36 patients (19 pediatric and 17 adult patients) and showed no significant correlation with MSTN levels. Similarly, no significant correlations with MSTN were observed for body mass index (BMI), available in 23 patients (7 pediatric and 16 adult patients), or creatinine levels, available in 34 patients (19 pediatric and 15 adult patients). Follow‐up data for CK, creatinine, and BMI were too limited to allow meaningful statistical analyses. Changes in body weight during follow‐up were available for 24 patients (12 pediatric and 12 adult patients) and were not significantly correlated with changes in MSTN levels.

### 3.3 MSTN and Smad3 Levels in Serum of Pediatric and Adult SMA Patients During Therapy

Statistical analysis did not reveal significant changes in serum MSTN and Smad3 levels in both pediatric and adult SMA patients during the treatment period. However, considering the FC values (i.e., ratio between post‐treatment and baseline values; FC > 1 indicating an increase, FC < 1 a decrease and FC = 1 no change), MSTN was reduced in 5/12 (41.7%) and Smad3 in 7/12 (58.3%) patients in the pediatric group. On the other hand, MSTN was increased in 6/12 (50%) and unchanged in 1/12 (8.3%) pediatric patient; Smad3 was increased in 4/12 (33.3%) and unchanged in 1/12 (8.3%) pediatric patient. In the adult cohort, MSTN was reduced in 9/18 (50%) and Smad3 in 11/18 (61.1%) patients and resulted increased in 9/18 (50%) and 7/18 (38.9%) patients, respectively. Notably, Smad3 resulted reduced in all adult and pediatric non‐sitter patients and MSTN resulted increased in all non‐sitter adult patients; no pediatric non‐sitter patient was included in the whole cohort (Table 3).

**TABLE 3:** Decreased and increased Myostatin and Smad3 levels, according to FC values, in pediatric and adult SMA patients.

	Non sitter	Sitter	Walker
PEDIATRIC			
↓ MSTN	/	4/5 (80%)	1/5 (20%)
↓ Smad3	6/7 (85.7%)	1/7 (14.3%)	/
ADULT			
↓ MSTN	/	3/9 (33.3%)	6/9 (66.7%)
↓ Smad3	2/11 (18.2%)	2/11 (18.2%)	7/11 (63.6%)
PEDIATRIC			
↑ MSTN	/	5/6 (83.3%)	1/6 (16.7%)
↑ Smad3	/	2/4 (50%)	2/4 (50%)
ADULT			
↑ MSTN	2/9 (22.2%)	1/9 (11.1%)	6/9 (66.7%)
↑ Smad3	/	2/7 (28.6%)	5/7 (71.4%)

Abbreviation: MSTN, Myostatin.

To verify whether the observed changes in serum MSTN and Smad3 levels were influenced by treatment type, the cohort was stratified into SMA patients receiving either nusinersen or risdiplam. No significant differences in MSTN or Smad3 levels were observed between the two treatment groups. In addition, statistical analyses did not reveal a significant correlation between MSTN and Smad3 expression levels across different time points in pediatric and adult groups (data not shown). No significant correlation was found between FC values of MSTN and Smad3 and responder status.

### 3.4 Correlation Between Serum MSTN and Smad3 Levels and HFMSE Motor Scale of SMA Patients During Treatment

The analyses in the entire cohort revealed a significant positive correlation between serum MSTN levels and HFMSE motor scale both at baseline (*r* = 0.56, *p* = 0.0004) and post‐treatment (*r* = 0.60, *p* = 0.0006) (Figure 2A,B); no significant correlations were observed based on FC values. For Smad3, no significant correlations were identified at any time point.

**FIGURE 2:**
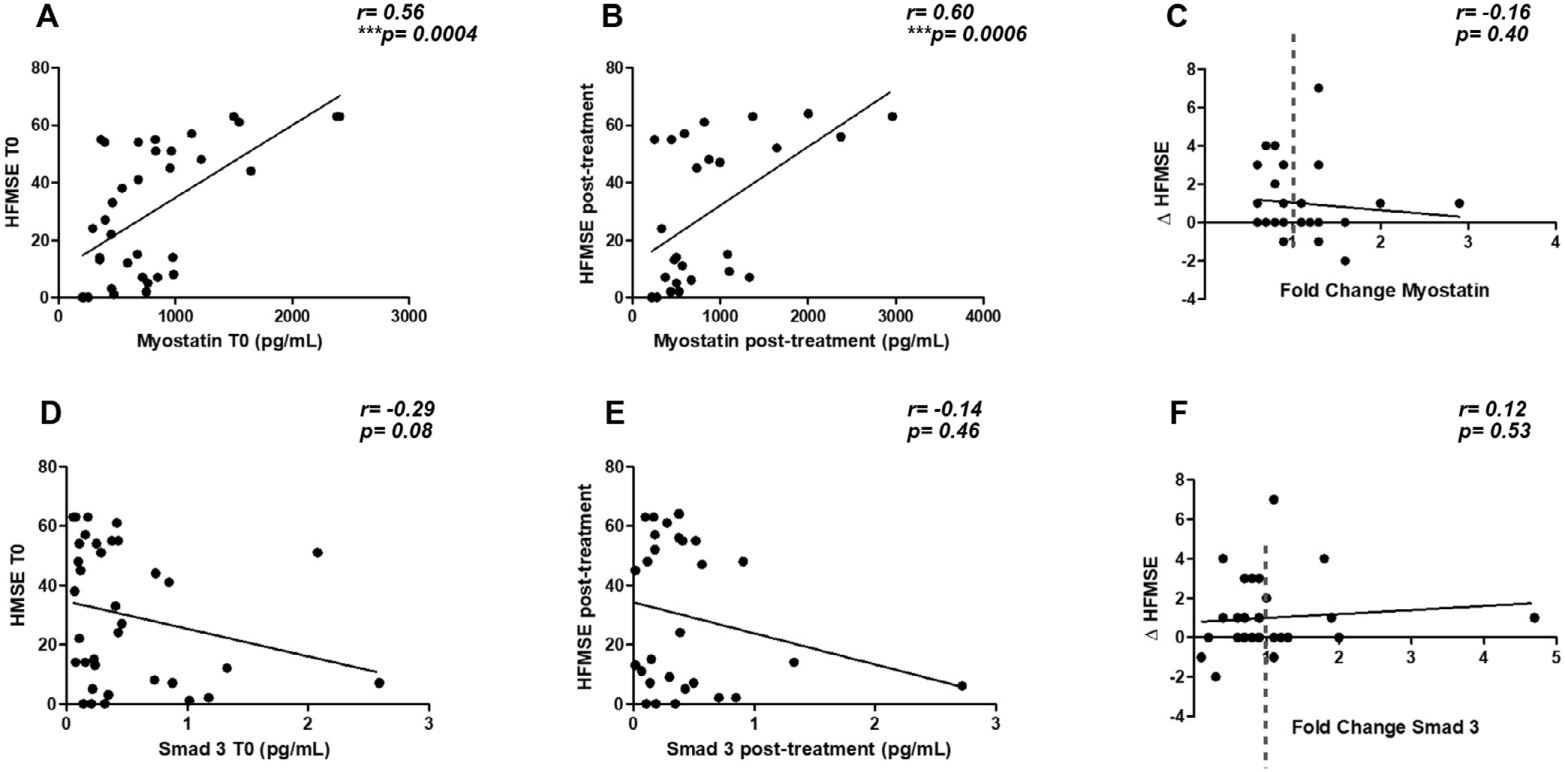
Spearman's correlation coefficients (r) were used to corroborate relationships between serum MSTN or Smad3 levels and HFMSE motor scale in SMA patients at baseline (T0) (A, D), after treatment (B, E), and based on Fold change (FC) (C, F). FC was calculated as the ratio between post‐treatment and baseline values; FC > 1 indicates an increase, FC < 1 indicates a decrease, and FC = 1 indicates no change. Correlation coefficients greater than 0.5 indicate a good positive correlation.

Subsequently, the cohort was stratified into pediatric and adult subgroups to assess whether age‐related differences existed in the correlation between serum MSTN and Smad3 levels and HFMSE motor scores. Of interest, in the pediatric cohort, a significant negative correlation was found between serum MSTN levels and HFMSE motor scale based on FC values (*r* = −0.75, *p* < 0.008, Figure 3C), whereas no significant correlations were observed at baseline and T6, respectively. Moreover, a significant negative correlation (*r* = −0.50, *p* < 0.03) was observed between Smad3 levels and HFMSE motor scale at baseline (Figure 3D); conversely, no significant associations were detected at T6 and based on FC values.

**FIGURE 3:**
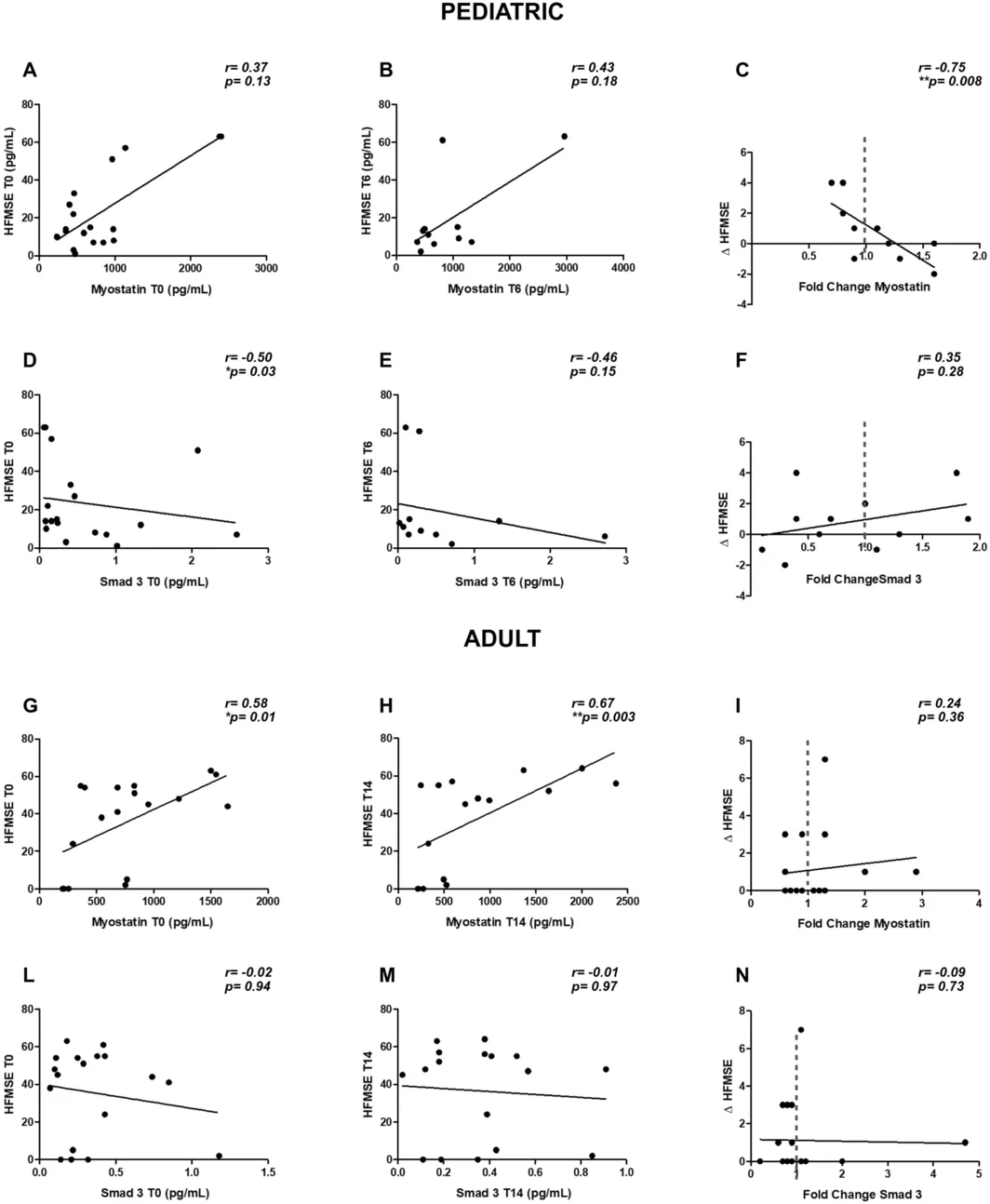
Spearman's and Pearson's correlation coefficients (*r*) were used to corroborate relationships between serum MSTN or Smad3 levels and HFMSE motor scale at baseline (A, D, G, L), after treatment (T6 for pediatric and T14 for adult SMA patients) (B, E, H, M), and based on fold change (FC) (C, F, I, N). In pediatric patients (A–F), panels A–C show correlations of HFMSE motor scale with serum MSTN levels, whereas panels D–F show correlations with serum Smad3 levels. In adult patients (G–N), panels G–I show correlations of HFMSE motor scale with serum MSTN levels, whereas panels L–N show correlations with serum Smad3 levels. FC was calculated as the ratio between post‐treatment and baseline values; FC > 1 indicates an increase, FC < 1 indicates a decrease, and FC = 1 indicates no change. Correlation coefficients greater than 0.5 indicate a good positive correlation.

Regarding the adult cohort, a significant positive correlation was identified between serum MSTN levels and HFMSE motor scale both at baseline (*r* = 0.58, *p* < 0.011, Figure 3G) and T14 (*r* = 0.67, *p* < 0.003, Figure 3H), while no significant correlation was observed based on FC values. Moreover, no significant correlation was found for Smad3 at any time point in the adult cohort.

### 3.5 Correlation Between Serum MSTN and Smad3 Levels and RULM Motor Scale of Pediatric and Adult SMA Patients During Treatment

In the pediatric cohort, a significant negative correlation was observed between serum Smad3 levels and the RULM motor scale at T6 (*r* = −0.65, *p* < 0.03, Figure 4E). A trend was noted at baseline between Smad3 levels and the RULM motor scale (*r* = −0.45, *p* = 0.05, Figure 4D), although no significant correlation was detected based on FC values. Furthermore, no significant correlations were observed between MSTN levels, also when considering FC values, and the RULM motor scale across the different time points. Regarding the adult cohort, analyses did not reveal any significant correlation between the RULM motor scale and either MSTN or Smad3 levels across the assessed time points.

**FIGURE 4:**
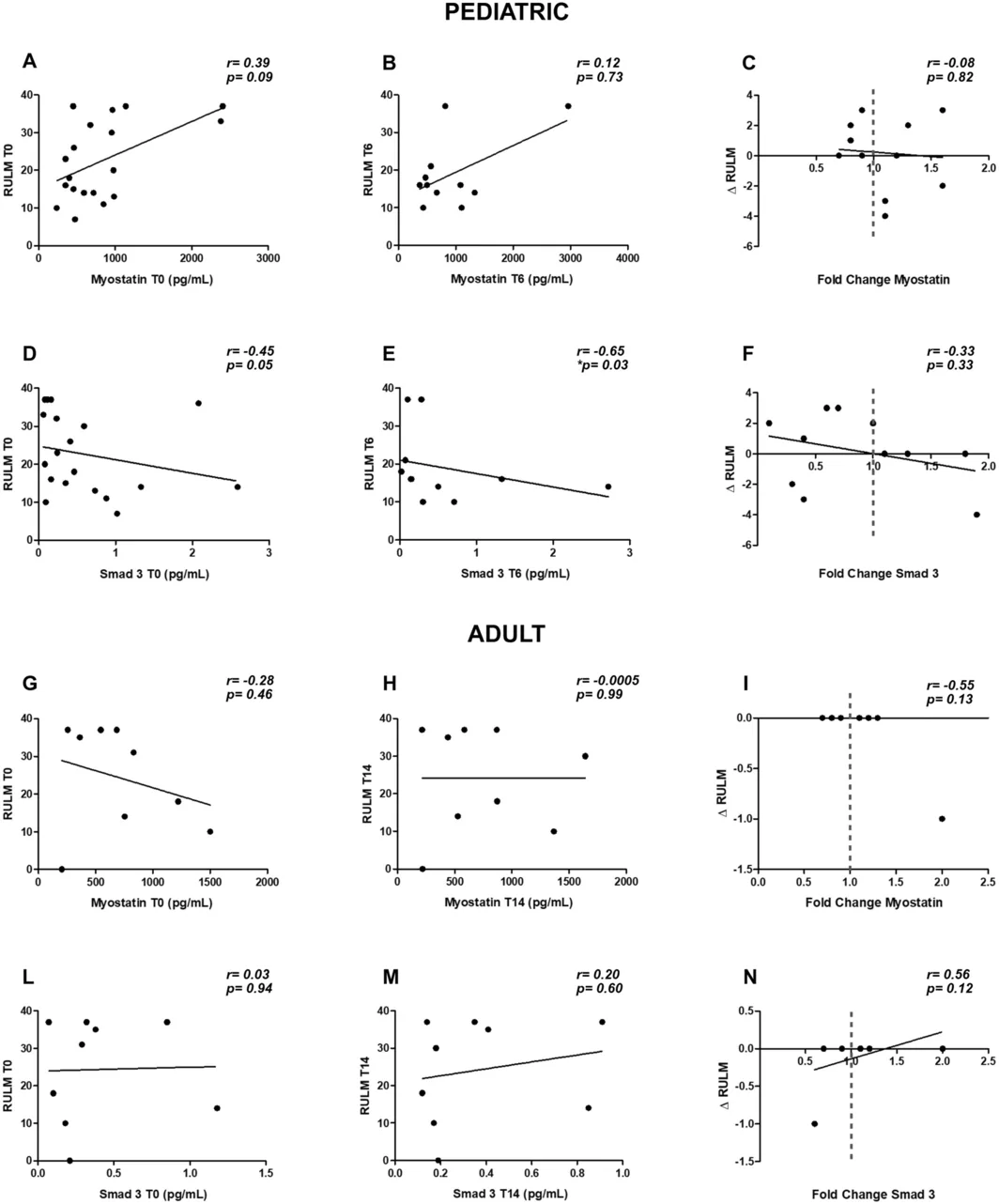
Spearman's and Pearson's correlation coefficients (*r*) were used to corroborate relationships between serum MSTN or Smad3 levels and RULM motor scale at baseline (A, D, G, L), after treatment (T6 for pediatric and T14 for adult SMA patients) (B, E, H, M), and based on fold change (FC) (C, F, I, N). In pediatric patients (A–F), panels A–C show correlations of RULM motor scale with serum MSTN levels, whereas panels D–F show correlations with serum Smad3 levels. In adult patients (G–N), panels G–I show correlations of RULM motor scale with serum MSTN levels, whereas panels L–N show correlations with serum Smad3 levels. FC was calculated as the ratio between post‐treatment and baseline values; FC > 1 indicates an increase, FC < 1 indicates a decrease, and FC = 1 indicates no change. Correlation coefficients greater than 0.5 indicate a good positive correlation.

## 4 Discussion

Over the past years, several biomarkers have been identified for SMA. However, despite their contribution to improving our understanding of the disease pathophysiology and treatment monitoring, they still present limitations that restrict their applicability in clinical and research practice [7, 24, 25]. In this context, MSTN has been investigated as a promising biomarker due to its central role in the negative regulation of muscle mass and its involvement in signaling pathways dysregulated in SMA [15, 16, 26]. Our study provides novel insights on MSTN as a potential biomarker of treatment response, extending for the first time the investigation to Smad3, given their close functional relationship within the same signaling pathway, in SMA patients receiving DMTs.

At baseline, MSTN levels are significantly increased in patients with SMA III compared to II and in walker compared to non‐sitters SMA patients, but not versus sitters. This pattern suggests that higher MSTN levels are associated with milder SMA phenotypes, likely reflecting greater preservation of muscle mass, in line with the established physiological role of MSTN as a negative regulator of skeletal muscle mass. These findings are in agreement with previous studies, reporting a positive correlation between MSTN expression and residual muscle mass in SMA patients, supporting its potential use as a biomarker for disease severity and functional status [15, 16, 27]. Similar associations have been reported also in non‐neuromuscular conditions, where MSTN levels correlate inversely with muscle mass and strength [28]. Moreover, the significant positive correlation at baseline between MSTN levels and HFMSE scores in adult patients, including mainly SMA III, further supports this relationship.

In our cohort, males showed significantly higher MSTN levels in comparison with females, aligning with clinical evidence of a gender effect in SMA disease manifestation and suggesting that sex‐related factors such as muscle metabolism, hormonal milieu, or compensatory mechanisms may influence MSTN regulation [29]. Notably, these higher MSTN levels were not associated with differences in disease severity, as HFMSE and RULM scores did not differ significantly between males and females at baseline. This observed gender effect on MSTN levels is in agreement with Piemonte et al.; conversely, a previous study by Albuquerque reported no difference between males and females, probably due to discrepancies among the 2 cohorts, among which age at baseline was higher in our population (22 vs. 11 years). Of note in healthy individuals, circulating MSTN levels may differ between males and females, with some studies reporting higher levels in men while others found no significant sex differences [30]. Collectively, these findings suggest that gender could represent an additional factor influencing MSTN expression in SMA, and its potential role in disease modulation should be further explored.

Conversely, and in line with other studies, no significant associations were found between baseline MSTN levels and age, disease duration, or *SMN2* copy number, suggesting that serum concentrations of these proteins may nevertheless reflect functional status, regardless of genetic background or disease duration [16, 26]. Similarly, Piemonte and colleagues found no correlation between MSTN and either age or disease duration. However, in the same study, they reported significant differences in MSTN levels according to *SMN2* copy number, indicating that a higher number of *SMN2* copies is associated with increased MSTN levels [15]. This difference may be in relation to the predominant inclusion of patients with 3 or 4 *SMN2* copies in our cohort, whereas one third of the patients included in the study by Piemonte et al. had 2 *SMN2* copies.

Regarding baseline Smad3 levels, no significant differences were observed across gender, age, SMA type, motor function, disease duration, or *SMN2* copy number. In addition, no correlation was found between MSTN and Smad3 levels across different time points. These findings may reflect that Smad3 is not exclusively regulated by MSTN and can also be activated by other TGF‐β superfamily ligands, such as TGF‐β1 and activins, which are involved in inflammation, immune regulation, and processes that reduce satellite cell activation, impair muscle regeneration, and promote fibrotic remodeling [31].

These biological aspects should be considered when interpreting circulating Smad3 measurements. Unlike MSTN, which is a secreted myokine that can be directly released into the circulation, Smad3 is primarily an intracellular signaling mediator, and its serum levels may not accurately reflect its activation status, tissue‐specific activity, or downstream transcriptional effects in skeletal muscle. Furthermore, circulating Smad3 concentrations may be influenced by multiple systemic processes beyond skeletal muscle biology, including inflammatory and fibrotic pathways, potentially reducing its specificity as a muscle‐related biomarker. Therefore, our findings do not support the use of serum Smad3 as a clinically applicable biomarker at this stage but rather suggest that further investigations, including analyses of Smad3 activation status, phosphorylated Smad3, and tissue‐specific signaling pathways in larger cohorts, are required to clarify its potential relevance in SMA.

Consistent with this interpretation, the negative correlation observed in the pediatric cohort between baseline Smad3 levels and HFMSE scores and between Smad3 and RULM scores at T6 suggests that higher Smad3 activity may be associated with a more compromised motor function, possibly reflecting a pro‐fibrotic and muscle inhibitory milieu [32]. This association, limited to pediatric patients, may be related to greater muscle plasticity and ongoing remodeling typical of early disease stages; whereas in adults, established structural alterations could attenuate the relationship between Smad3 signaling and functional measures.

Following treatment with currently available DMTs, our study provides novel insights into the dynamics of MSTN and, for the first time, Smad3 expression in SMA patients. Notably, MSTN FC showed significant negative correlation with HFMSE changes during the treatment period in pediatric patients. This association does not appear to be age‐related, as no significant correlation was found between age and MSTN levels but may rather reflect differences in clinical phenotype and disease severity, considering that most pediatric patients were affected by SMA II. Interestingly, patients showing improvement in HFMSE scores exhibited a reduction in MSTN levels independently of absolute baseline values. Given the role of MSTN as a negative regulator of muscle growth, this finding may suggest a dynamic treatment‐related response, potentially associated both with earlier therapeutic intervention and with clinical features characteristic of SMA Type 2. No further correlations between MSTN and Smad3 levels and clinical outcomes were observed in either pediatric or adult SMA cohorts during treatment, despite clinical meaningful response in around 60% and 30% of pediatric and adult patients, respectively; these findings were likely due to the high variability of these molecules over time, including both increases and decreases, and are similar to those reported by other studies, with relevant modifications limited to presymptomatic patients starting treatment during the neonatal period, in whom MSTN levels at baseline were reported to be similar to the controls [15, 26]. Interestingly, MSTN and Smad3 FC were consistently increased and decreased, respectively, across all pediatric and adult non‐sitter patients, except for MSTN in the pediatric subgroup where no non‐sitter patient was included. This further supports lack of correlation between the two biomarkers and suggests a potential beneficial effect in patients with more severe disease. However, these findings should be interpreted with caution given the small sample size.

Adult patients exhibited a significant correlation between HFMSE and MSTN levels at baseline and at the end of the treatment period; however, no association was observed between changes in HFMSE and MSTN FC during therapy or between RULM and MSTN levels in adult patients. Similarly, no correlation was found between MSTN levels and HFMSE or RULM at baseline and T6 in pediatric patients. These findings reflect the higher proportion of SMA III individuals in the adult cohort, who typically retain greater residual motor function and are therefore more appropriately assessed using the HFMSE. In contrast, the RULM is more suitable for evaluating more severely affected patients, such as those with SMA II, who constitute the majority of the pediatric cohort. Therefore, these results suggest that in adults MSTN may primarily reflect disease severity by HFMSE and residual muscle mass rather than serving as dynamic indicators of response to SMN‐targeted therapies [16].

Interestingly, no association was observed between MSTN or Smad3 changes under treatment and responder status. Moreover, the observed changes of MSTN and Smad3 were independent of treatment type, as no significant differences in MSTN or Smad3 modulation were noted between risdiplam‐ and nusinersen‐treated patients, in agreement with previously published studies on MSTN [15].

The differences observed between pediatric and adult SMA patients in biomarker associations and clinical outcomes warrant careful interpretation, as they may reflect the interplay of multiple disease‐related clinical factors, rather than be interpreted as evidence of age‐dependent biological mechanisms regulating MSTN or Smad3 dynamics. In particular, differences in disease stage, SMA subtype distribution, baseline motor function, residual muscle mass, disease duration, treatment timing, and therapeutic response may contribute to the distinct patterns observed between pediatric and adult cohorts. Therefore, future studies including larger, age‐balanced cohorts with longitudinal follow‐up will be required to disentangle the contribution of age from other disease‐related factors and to further clarify the role of these biomarkers across the SMA spectrum.

Overall, our findings support the role of MSTN as a biomarker of disease severity and functional status in adult and pediatric SMA patients, respectively. Moreover, our data suggest a potential role of MSTN as a dynamic biomarker for monitoring disease progression in pediatric patients, regardless of the ongoing treatment. Conversely, the role of Smad3 as a biomarker in SMA appears more limited and requires further investigation. Future studies should clarify which form of Smad3 is most informative, including its phosphorylated/activated form, as total circulating Smad3 may not accurately reflect tissue‐specific Smad3 activity or downstream signaling.

Indeed, the main limitation of this study is the relatively small sample size and the heterogeneous composition of the cohort, which may contribute to the inter‐individual variability observed in some parameters and potentially limit the statistical power of subgroup analyses. Nevertheless, similar studies investigating SMA biomarkers or therapeutic outcomes often involve relatively small cohorts due to the rarity of the disease and the challenges associated with patient recruitment [16, 24, 26]. Therefore, the present study should be considered exploratory and hypothesis‐generating. Another limitation of this study is the lack of pediatric healthy controls due to difficulty in collecting biological samples for the biobank in healthy children. This restricts the information we can gather about MSTN and Smad3 baseline expression levels.

Despite these limitations, our findings provide valuable insights into the potential role of circulating MSTN in SMA pathophysiology and treatment monitoring. Future studies in larger, independent, and ideally multicenter longitudinal cohorts will be essential to validate these observations and to further define the response patterns of MSTN and related biomarkers across different DMTs.

## Author Contributions

L.M. and G.R. designed the study and drafted the manuscript. G.R. performed the experiments and conducted the data analyses. G.R., L.M., S.B., S.M., and R.M. interpreted the results. M.C., E.G.G., C.D., S.P., and R.Z. were responsible for clinical data collection. F.A. and A.C. collected the biological samples and contributed to the experimental procedures. All authors critically revised the manuscript and approved the final version.

## Funding

This work was partially supported by the Italian Ministry of Health (RRC), Innova plus, and the Regional Foundation for Biomedical Research (FRRB) under Grant No. 4956734.

## Conflicts of Interest

L.M. received honoraria for speaking, advisory boards and compensation for congress participations from Sanofi, Roche, Biogen, Amicus Therapeutics, Alexion Astrazeneca, Johnson & Johnson, Lupin, Argenx, and UCB, outside the submitted work. L.M. also received funding for fellowship from Biogen and Alexion Astrazeneca. S.B. received honoraria for speaking, advisory boards and compensation for congress participations from Sanofi, Roche, Biogen, Alexion Astrazeneca, and UCB. R.M. received honoraria for speaking and advisory boards from Biogen, Roche, Novartis, PTC, Italfarmaco, Santhera, and Pfeizer. C.D., S.P., and R.Z. received honoraria for speaking and compensation for congress participations from Roche, Biogen, and Novartis. M.C. received honoraria for speaking and compensation for congress participations from Roche, Alexion, and Argenx. E.G.G. received honoraria for participation in advisory boards for Amicus Therapeutics. G.R., S.M., F.A., and A.C. declare no conflicts of interest.

## Data Availability

The data that support the findings of this study are available from the corresponding author upon reasonable request.
